# Productivity and production risk effects of adopting drought-tolerant maize varieties in Zambia

**DOI:** 10.1108/IJCCSM-03-2018-0024

**Published:** 2019-08-19

**Authors:** Emily Amondo, Franklin Simtowe, Dil Bahadur Rahut, Olaf Erenstein

**Affiliations:** 1 Centre for Development Research (ZEF), University of Bonn, Bonn, Germany; 2 International Maize and Wheat Improvement Center (CIMMYT), Nairobi, Kenya; 3 International Maize and Wheat Improvement Center (CIMMYT), Texcoco, México

**Keywords:** Risk, Productivity, Endogenous switching regression, Drought tolerance, Improved maize varieties

## Abstract

**Purpose:**

Productivity and production risks affect the use of agricultural production practices and inputs, particularly in developing countries. This paper aims to investigate the effects of adopting drought-tolerant maize varieties (DTMVs) on farm productivity, yield variance and downside risk exposure of maize growing households of Zambia.

**Design/methodology/approach:**

The study uses household survey data collected from 11 maize producing districts of Eastern, Southern and Copperbelt provinces of Zambia using a structured questionnaire. The Antle’s flexible moment-based approach was used in specifying, estimating and testing a stochastic production function. The study further applied an endogenous switching regression model to control for both observable and unobservable sources of bias.

**Findings:**

The study revealed that DTMV adoption increases maize yield by 15 per cent and reduces the risk of crop failure: reducing yield variance by 38 per cent and exposure to downside risk by 36 per cent.

**Originality/value:**

This study establishes the benefits of DTMV adoption in Zambia with regards to productivity, yield stability and downside risk in the face of climate change. Results from this study underscore the need for more concerted efforts to scale-out DTMVs for both maize productivity enhancement and for risk mitigation against weather shocks.

## 1 Introduction

Farm households in Sub-Saharan Africa (SSA) typically rely on rain-fed agriculture which exposes them to the risks of low productivity and crop failure resulting from weather shocks. Besides, low level of input use, lack of finances and infrastructure, lack of adequate knowledge on best management practices and the frequent incidence of pests and diseases, exacerbate the impacts of climate change in developing countries (Yohe and Tol, 2002; Mirza, 2003; McSweeney *et al*., 2010). These constraints have serious implications not only on farm productivity but also on food security and other welfare indicators at both household and national levels. The impacts of climate change on agriculture thereby notably affect the rural poor who are least able to adapt, and this adds significantly to the development challenges of ensuring food security and reducing poverty (Jones and Thornton, 2003).

Drought risk, in particular, is a major concern since it has serious and complex economic, social and environmental implications for rural communities [Food and Agricultural Organizations of the United Nations (FAO), 2015a, 2015b; Monacelli *et al*., 2005]. In SSA, droughts and floods alone are estimated to account for 80 per cent of the loss of life and 70 per cent of the economic losses (Bhavnani *et al*., 2008; Shiferaw *et al*., 2014; Hlalele *et al*., 2016). Drought vulnerability and impacts in SSA are further aggravated by population growth, poverty and inadequate policies (Tadesse, 1998; Shiferaw *et al*., 2014).

At the center of the drought challenge in SSA is maize: the continent’s most important staple food crop, consumed by 50 per cent of the population, yet susceptible to drought (CRP MAIZE[1]; Sangoi and Salvador, 1998; Aslam *et al*., 2015). In Zambia, maize occupies a central position in its agricultural political economy as both the national staple food and primary smallholder crop (Chapoto *et al*., 2015). While the country ranked 13th among the 51 maize producing African countries with a total production of 0.865 million tons of maize in 2006 [Japan Association for International Collaboration of Agriculture and Forestry (JAICAF), 2008], a tremendous increase in maize production has been observed since with an estimated 3.607 million tons in 2016 (Chapoto *et al*., 2017). The increase is largely due to area expansion and increased government spending in the maize sector (Chamberlin *et al*., 2014). Nearly a third of the total arable land was under maize production in 2011/2012. Zambia spends 50-80 per cent of its agriculture budget on input and output subsidies through its Farmer Input Support Program (FISP) and Food Reserve Agency in the quest to achieve national maize security objective (Chapoto *et al*., 2015; Kuteya *et al*., 2017).

**Figure 1:**
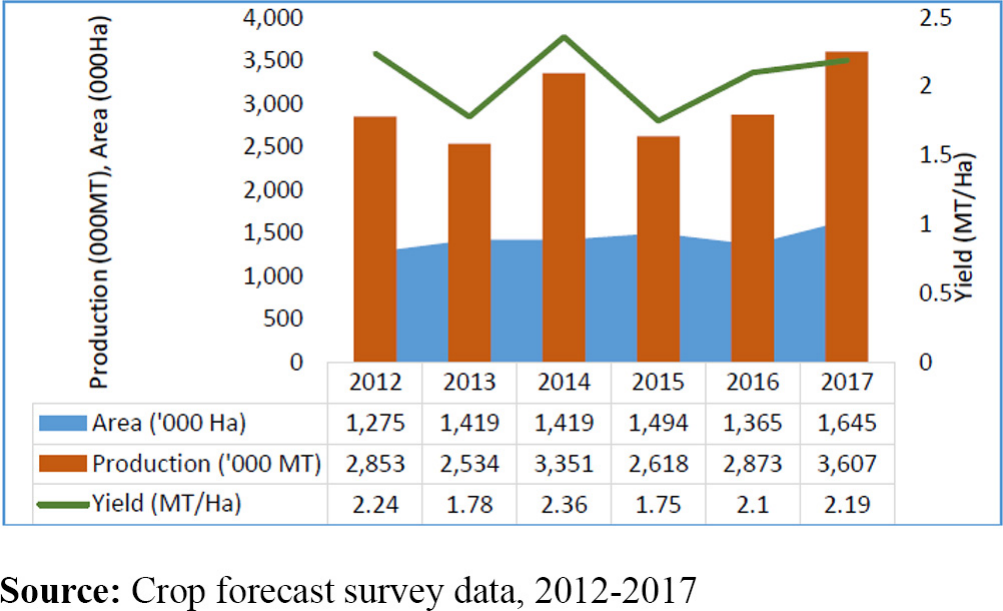
Maize production trends in Zambia

Despite the importance of maize and the concerted efforts by government in the maize sector in Zambia, the country continues to battle with low and variable maize productivity (oscillating around 2 tons per hectare as affected by drought, Figure 1; as compared to the worldwide average of 5.5 tons) and high rates of rural poverty 77 per cent (Republic of Zambia – Central Statistical Office, 2016; Chamberlin *et al*., 2014). Among the ten Zambian provinces, the Eastern province is the largest maize producer, followed by the Southern and Central provinces (JAICAF, 2008; Chamberlin *et al*., 2014). Zambian communities are vulnerable to weather hazards, as they rely solely on rain-fed agriculture and lack the capacity, resources and financial assistance to adapt to and overcome worsening climatic conditions [Government of Zambia and United Nations Development Program (GRZ and UNDP), 2010]. Prolonged dry spells and shorter rainfall seasons over the past 20 years have been associated with reduced maize yields to 40 per cent of the long-term average [Government of Zambia and United Nations Development Program (GRZ and UNDP), 2010]. Ngoma (2008) reports rapidly increasing temperatures in Zambia (0.6 degrees Celsius per decade), at rates higher than those of Southern Africa (0.5°C). A projected change in Africa’s annual mean temperature above 2°C in the mid-twenty-first century as compared to late twentieth century levels will adversely affect maize yields and food security in future [Intergovernmental Panel on Climate Change (IPCC), 2014].

African maize farming households deploy different coping strategies to cope with weather risks and adapt to climate change (Dercon, 2004; Fisher *et al*., 2015). Nonetheless, some of these methods are insufficient for protecting livelihoods in drought-prone regions of SSA (Shiferaw *et al*., 2014; Fisher *et al*., 2015). One effective, innovative adaptation strategy is the use of drought-tolerant maize varieties (DTMVs). These varieties have been developed since 2006 and deployed to over 13 countries in Eastern, West and Southern Africa and beyond, by the International Maize and Wheat Improvement Center (CIMMYT) in collaboration with other CGIAR centers, National Research Institutions and seed producers (Shiferaw *et al*., 2014; Fisher *et al*., 2015; Wossen *et al*., 2017). DTMVs are well-adapted to SSA and include hybrids and open-pollinated varieties. DTMVs are expected to increase farmers’ maize yields by at least one ton per hectare under moderate drought and increase farmers’ yields by 20-30 per cent, reduce yield variability and reduce production risk (CIMMYT-DTMA[2]; Shiferaw *et al*., 2014; Wossen *et al*., 2017). The DTMVs are not only tolerant to drought but some also possess desirable traits such as resistance to major diseases, superior milling or cooking quality and/ or better nitrogen use efficiency (CIMMYT-DTMA[2]; Fisher *et al*., 2015; Wossen *et al*., 2017).

This paper contributes to the emerging body of literature by documenting the productivity and production risks effects of adopting DTMVs in Zambia using the Antle’s moment-based approach (mean yield, variance and skewness). Despite several previous studies on adoption, productivity, production risk and welfare impacts of different agricultural technologies, to-date there is no study assessing the impacts of DTMVs in Zambia. Previous studies focused on determinants of DTMV adoption (Fisher *et al*., 2015) and ex-ante assessment of the potential impacts in investing in DTMVs in Africa (La Rovere *et al*., 2014; Kostandini *et al*., 2013). Our analysis uses an endogenous switching regression approach to control for both observed and unobserved sources of heterogeneity. The rest of the paper is organized as follows. In Section 2, a brief review of the literature on farm technology adoption and impact is presented. Materials and methods are outlined in Section 3. Section 4 presents and discusses the descriptive and econometric results. Conclusion and policy implications are presented in Section 5.

## 2 Literature review

Production and consumption risks play a critical role in the choice and use of agricultural production practices and inputs in countries where insurance and credit markets are thin or missing (Yesuf *et al*., 2009; Juma *et al*., 2009). Production risks play a key role in agricultural production decisions and can worsen social welfare in the absence of mechanisms that serve to minimize its downside effects (Antle, 1983, 1987; Dercon, 2004; and Kassie *et al*., 2008). Productivity and risk implications vary by technology, and this plays a non-trivial role in the farmer’s adoption decision, especially in low-income, rain-fed agriculture (Yesuf *et al*., 2009; Kassie *et al*., 2008; Juma *et al*., 2009). Apart from increasing productivity and welfare, the adoption of new farm technology could increase production risk either by increasing yield variability or by increasing the probability of crop failure, or vice versa (Yesuf *et al*., 2009). Some of the past studies on the impacts of different technologies on productivity, production risk and welfare are detailed below.

Juma et al. (2009) assessed the production risks and farm technology adoption among smallholder farmers in Kenya and noted that, among others, yield variability and the risk of crop failures indeed affect technology adoption decisions in low-income, rain-fed agriculture though the direction and the magnitude of the effects depends on the farm technology under consideration. Their results indicated that the first moment had a highly significant positive effect on fertilizer adoption and manure application while yield variability had a negative impact on manure application, intensity of manure and fertilizer application. Results on downside risk showed that a higher probability of crop failure increases the farmers’ chance of adopting terracing and reduces the possibility of fertilizer adoption. Their study concluded that productivity gains are necessary, but not sufficient, conditions to attract farmers to adopt new technologies and agricultural innovations. Poor farm households in rain-fed and risky production environments are often reluctant to adopt new farm technologies with potential production gain because, at the same time, they may increase downside risks (Juma *et al*., 2009).

A study by Kassie et al. (2008) applied a moment-based approach to assess the role of production risk on the adoption of sustainable land-management technology in Ethiopia. They found that while expected return had a positive and significant impact on both chemical fertilizer (adoption and intensity) and conservation adoption, production risks had a significant impact on only fertilizer adoption and the extent of adoption and, no statistical significance impact on the adoption of the conservation technology. On the other hand, Yesuf et al. (2009) report that chemical fertilizer adoption reduced yield variability, but increased the risk of crop failure while the adoption of soil and water conservation technology had no impact on yield variability, but reduced the downside risk of crop failure. The implications from these studies is that the impact of production risk varies by technology type and call for the combined assessment of implications for variance of return and exposure to downside risk, productivity and food production.

DTMV adoption in SSA is expected to generate substantial benefits to both producers and consumers, and DTMVs may be an effective tool for reducing household risk, especially for the poor who face high drought risk and are highly dependent on cereal production (Kostandini *et al*., 2013; La Rovere *et al*., 2014). DTMV adoption could generate cumulative benefits of US$362-590m to both producers and consumers across SSA by 2016 (Kostandini *et al*., 2013). They further observed that the role of DTMVs in variance reduction accounts for a significant share of total benefits and translate into poverty reductions. Specifically, yield variance reduction accounted for 10 per cent of the total benefits, and it was noted that the risk benefits (variance reduction) appear to be more important in more drought-prone areas. These ex-ante studies suggested that policies and investments that set up the right infrastructure for the production and dissemination of DTMVs may prove to be very beneficial in both the short and long term.

A recent study by Wossen et al. (2017) explored the impacts of DTMVs on productivity, welfare and risk exposure in Nigeria. They found out that the adoption of DTMVs indeed increased maize yields by 13 per cent and reduced the level of variance by 53 per cent and downside risk exposure by 81 per cent among adopters. As a result, there was a reduction of 13 per cent in poverty incidence and 84 per cent in the probability of food scarcity among adopters. From this study, it is inferred that, interventions against drought stress through genetic improvements and the subsequent adoption of these improved technologies will have a critical role to play in terms of enhancing food security and reducing farmers’ exposure to drought risk (Wossen *et al*., 2017).

Khonje et al. (2015) conducted a study of 810 households from eastern Zambia in 2012 and documented the welfare impacts of improved maize varieties (IMVs). Analysis revealed that adoption of IMVs generates significant benefits in crop incomes, consumption expenditure and food security. An average increase in crop income per hectare that ranged from (US$15) using the endogenous switching regression (ESR) technique to US$455 of the propensity score matching (PSM) was evident. PSM showed an average consumption expenditure per capita of US$52-59 and a reduction in the probability of poverty by 11 per cent points. While ESR results showed a higher impact on consumption expenditure per capita of US$62 and reduction in the probability of poverty by 21 per cent points for adopters. A further study by Manda et al. (2016) on the impact of three sustainable agricultural practices (SAPs) including IMVs on the same data set revealed that SAPs adopted in combination had a strong and positive impact on maize yields and household income compared to those adopted in isolation, except for the adoption of IMVs. Adoption of IMVs alone had greater impacts on maize yields (90 per cent) while adoption of a more comprehensive package consisting of the three SAPs resulted in the yield effect of 80 per cent and an increase in income per capita of 43-75 per cent.

## 3 Materials and methods

### 3.1 Conceptual framework

Decision-making process under risk and uncertainty is usually a challenging task to the farmer. Nevertheless, this process is an integral part of planning, and sound decisions need to be made for optimum farm production. A good production technology in combination with other complementary inputs is needed for a farm producing output y under risk so as to enable a farmer maximize the expected utility of net returns from the production. The flexible moment-based approach proposed by Antle (1983) was used in specifying, estimating and testing stochastic production function. Specifically, the approach was used to not only estimate mean output as a function of inputs but also second and third moments as functions of inputs. The moment-based approach provides a useful framework for testing the stochastic structure of production because it imposes relatively fewer restrictions (Antle, 1983).

The stochastic maize production function of a farmer producing maize output using several inputs under risk is specified as *y* = *g* (*m*, *x*, *w*), where *y* is maize output, *m* represents DTMV seed, *x* is a vector of other inputs other than maize seed and *w* is a vector of random variables representing uncontrollable factors affecting maize output such as rainfall whose insufficient amounts results into drought shock – this is the source of production risk and *g* (*m, x, w*) represents the equivalent production technology, given m, x and w.

Further to the above specifications, it is also assumed that a typical farmer acquires DTMVs seed (**m**) with a unit cost **c** and other inputs (**x**) with a unit cost (**t**). The prices **p** and cost of production c and t are assumed to be non-random as farmers are price takers in both input and output markets (Wossen *et al*., 2017). To capture the risk factors of production, we considered the variance and skewness of the maize yield by applying the moment-based approach where the higher moments of *g* (*m, x, w*) are given by:

(1) $$E\left[g\left(m,x,w\right)-f_{1}{\left(m,x,w\right)}^{k}\right]=f_{k}\left(m,x,w,\beta_{k}\right)\forall k\geq 2$$

where *f*_1_(.) = *E*(*y*) = *E*(*g*(*m, x, w*)) represents the mean of output. Usually, it is expected that the mean output to be increasing and concave in inputs – *m, x* (Di Falco and Chavas, 2009). Based on the above equation, the first moment (mean) of production function is therefore defined as:

(2) $$\mu_{1}=E\left[g\left(m,x,w\right)\right]=f_{1}\left(m,x,w,\beta_{1}\right)-cm-tx$$

Variance of the production represented by the second central moment μ2 is defined as:

(3) $$\mu_{2}=E\left[{\left(g\left(m,x,w\right)-E\left(g\left(m,x,w\right)\right)\right)}^{2}\right]$$

The production function third central moment (measuring skewness) is specified as:

(4) $$\mu_{3}=E\left[{\left(g\left(m,x,w\right)-E\left(g\left(m,x,w\right)\right)\right)}^{3}\right]$$

Di Falco and Chavas (2009) denote that the third central moment which considers the effects of skewness and downside risk exposure provides a flexible representation of the impacts of inputs on the distribution of output under production uncertainty.

Unlike in mean output, which is always expected to be on an increasing trend and concave to inputs, the effects of inputs *(m, x)* on variance and skewness of output is predominantly an empirical issue. The ith input could be variance increasing, variance neutral or variance decreasing or could be decreasing or increasing downside risk exposure (Di Falco and Chavas, 2009). In our setting, the effect of DTMVs on variance and skewness is of special interest, and as a risk mitigation strategy, DTMVs are expected to reduce both variance and downside risk.

For risk-averse farmers whose aim is to maximize the expected utility of net returns from maize production, he will adopt improved technology if the expected utility with adoption *E*[*u* (π^1^)] is greater than the expected utility without adoption *E*[*u*(π^0^)] (Kassie *et al*., 2008):

(5) $$E\left[u\left(\pi^{1}\right)\right]-E\left[u\left(\pi^{0}\right)\right]>0$$

The risk premium depends on all pertinent moments of the profit distribution, and there is usually a close relationship between the moments of income p and the corresponding moments of production *g* (*m, x, w*). Therefore, the equation for maximization of the expected utility of net returns from maize production for a risk-averse farmer is specified as:

(6) $$E_{\max}E\left[u\left(\pi \right)\right]=u\left(\mu_{1},\mu_{2},\mu_{3}\right)$$

Taking into consideration Taylor series of approximation of the risk premium the optimum condition of adopting DTMVs in elasticity form is given by:

(7) $$\left(\mu_{1}{}^{*}-\frac{cm}{\mu_{1}}\right)-\frac{1}{2}\left(\frac{u^{\prime \prime }\left(\pi \right)}{u^{\prime }\left(\pi \right)}m_{2}\right)U_{2}{}^{*}+\frac{1}{6}\left(\frac{u^{\prime \prime \prime }\left(\pi \right)}{u^{\prime }\left(\pi \right)}m_{3}\right)U_{3}{}^{*}=0$$

where $\mu_{1}{}^{*}-\frac{cm}{\mu_{1}}$ represents the marginal net return of choosing DTMVs (m^*^) and the two additive parts reflecting effect of variance *(m_2_)* and skewness *(m_3_)* $\left\{-\frac{1}{2}\left(\frac{u^{\prime \prime }\left(\pi \right)}{u^{\prime }\left(\pi \right)}m_{2}\right)U_{2}{}^{*}+\frac{1}{6}\left(\frac{u^{\prime \prime \prime }\left(\pi \right)}{u^{\prime }\left(\pi \right)}m_{3}\right)U_{3}{}^{*}\right\}$ represents the marginal risk premium of adopting DTMVs (Wossen *et al*., 2017; Di Falco and Chavas, 2009).

### 3.2 Empirical framework

The study applied the ESR model that not only accounts for observed sources of heterogeneity but also accounts for unobserved sources of bias. This is due to the assumption that there might be some unobservable farm or household variables that could possibly influence both adoption and outcome variables (Ahmed *et al*., 2017). With regards to the aforementioned conceptual framework, further assumption is that a specific farmer adopts DTMVs if the benefits expected from adoption (productivity gain and risk exposure reduction) are positive (benefit from adoption is greater than that of non-adoption). This means that a farmer will choose to adopt (A_i_ = 1) A^*^ > 0, 0 otherwise, where A^*^ represents the expected benefits of adopting with respect to not adopting. The selection equation for the latent variable (A_i_*) can thus be specified as follows:

(8) $$A_{\text{i}}{}^{*}=f\left(m,x,v,w,z,\gamma \right)+\mu_{1}\text{with}A=1\{A_{i}{}^{*}>0$$

where *m* refers to adoption of DTMVs seed, *x* represents other inputs other than DTMV seed, *v* refers to a vector of variables of socio-economic, farm and social capital, *w* is a vector of random variables representing uncontrollable factors affecting maize output such as rainfall, *z* is instrument variables (variables that affect the decision to adopt DTMVs but not the outcome indicators) and γ represents a vector of parameters to be estimated.

To account for selection bias, the ESR outcome equation conditional to DTMVs adoption – two regimes faced by the farmer are specified as follows:

(9) $$\text{Re}gime1:Y_{\text{l}i}=f\left(m,x,v,w,\beta_{1}\right)+\varepsilon_{\text{l}i}\text{if}A_{i}=1$$

(10) $$\text{Re}gime2:Y_{\text{2}i}=f\left(m,x,v,w,\beta_{2}\right)+\varepsilon_{\text{2}i}\text{if}A_{i}=0$$

where *Y_1i_* and *Y_2i_* are the dependent variables in the two continuous equations representing the yield of DTMVs adopters and non-adopters respectively. « _1i_ and « _2i_ are error terms of the outcome variables, and β _1_ and β _2_ are vectors of parameters to be estimated.

The error terms (μ _1_, « _1i_ and « _2i_) in both the selection equation (8) and outcome equations (9) and (10) are assumed to have a trivariate normal distribution, with zero mean and covariance matrix (X) as specified in the below equation:

$$\Omega =\left|\begin{array}{ccc} \sigma^{2}{{}_{\mu }}^{} & \sigma_{1\mu } & \sigma_{2\mu }\\ \sigma_{1\mu } & \sigma_{1}{}^{2} & \cdot \\ \sigma_{2\mu } & \cdot & \sigma_{2}{}^{2} \end{array}\right|$$

where $\sigma_{1}{}^{2}$ is a variance of the error term (μ _1_) in the selection equation, $\sigma_{1}{}^{2}$ and $\sigma_{2}{}^{2}$ are variances of the error terms (« _1i_ and « _2i_) the continuous outcome equations, σ _1u_ is the covariance of (μ _1_, « _1_), while σ _2u_ is the covariance of (μ _1_, « _2_). The covariance’s « _1i_ and « _2i_ are not defined as *Y_1i_* and *Y_2i_* are not observed simultaneously, and it is further assumed that and $\sigma^{2}{}_{\mu }$ is equal to 1 (Lokshin and Sajaia, 2011). If estimated covariance terms σ _1u_ and σ _2u_ are statistical significant, endogenous switching is evident and thus rejection of the null hypothesis of absence of sample selection bias (Di Falco *et al*., 2010; Wossen *et al*., 2017). Further estimation of inverse mill’s ratios (λ _1i_ and λ _2i_) computed from the selection equation (8) are included as auxiliary repressors in in equations (9) and (10) to correct for selection bias in the ESR, two-step estimation procedure.

#### 3.2.1 Conditional expectations, treatment and heterogeneity effects

In view of the above-mentioned ESR model, estimates of the average treatment effect on the treated households (ATT) and average treatment effect on the untreated households (ATU) are derived. These estimates allows comparison of the expected productivity gain and risk exposure reduction of the farm households that adopted DTMVs with respect to the farm households that did not adopt DTMVs and further investigate the expected productivity gain and risk exposure reduction in the counterfactual hypothetical cases that the adopted households did not adopt and that the non-adopted households adopted DTMVs. These four cases of conditional expectations for productivity in the four cases are specified as follows:

(11) $$E\left(Y_{1i}|A_{i}=1\right)=f\left(m,x,v,e,w,\beta_{1}\right)+{\text{λ}}_{\text{1}i}\sigma_{1\mu }$$

(12) $$E\left(Y_{2i}|A_{i}=0\right)=f\left(m,x,v,e,w,\beta_{2}\right)+{\text{λ}}_{\text{2}i}\sigma_{2\mu }$$

(13) $$E\left(Y_{2i}|A_{i}=1\right)=f\left(m,x,v,e,w,\beta_{2}\right)+{\text{λ}}_{\text{1}i}\sigma_{2\mu }$$

(14) $$E\left(Y_{1i}|A_{i}=0\right)=f\left(m,x,v,e,w,\beta_{1}\right)+{\text{λ}}_{\text{2}i}\sigma_{1\mu }$$

ATT and ATU of maize yield are therefore expressed as follows:

(15) $$ATU=E\left(Y_{1i}|A_{i}=1\right)-E\left(Y_{2i}|A_{i}=1\right)$$

(16) $$ATU=E\left(Y_{1i}|A_{i}=0\right)-E\left(Y_{2i}|A_{i}=0\right)$$

Heterogeneity effects were further estimated from the expected outcome equations. Effect of base heterogeneity for the sample group of farm households that decided to adopt DTMVs *(BH1)* was computed as the difference between equations (11) and (14), while effect of base heterogeneity for the sample group of farm households that decided not-to-adopt DTMVs *(BH2)* was computed as the difference between equations (13) and (12). Similarly, the transitional heterogeneity *(TH)* which evaluates whether the effect of adopting DTMVs is smaller or larger for the households that actually adopted DTMVs or the counterfactual case that they did not adopt was calculated as the difference between *ATT* and *ATU* (Table I).

In respect to our other objective of evaluating the effects of adopting DTMVs on risk exposure, the above equation specifications on ATT, ATU and heterogeneity effects were customized to reflect the effect on variance and skewness of maize yield as a measure of variability and downside risk (crop failure) as explained earlier in our moment-based approach. ATT and ATU for the second moment of maize yield (variance) were defined as follows:

(17) $$ATT=E\left(\mu_{1i}{}^{2}|A_{i}=1\right)-E\left(\mu_{2i}{}^{2}|A_{i}=1\right)$$

(18) $$ATU=E\left(\mu_{1i}{}^{2}|A_{i}=0\right)-E\left(\mu_{2i}{}^{2}|A_{i}=0\right)$$

Finally, ATU and ATT estimates for the third moment of maize yield were defined as:

(19) $$ATT=E\left(\mu_{1i}{}^{3}|A_{i}=1\right)-E\left(\mu_{2i}{}^{3}|A_{i}=1\right)$$

(20) $$ATU=E\left(\mu_{1i}{}^{3}|A_{i}=0\right)-E\left(\mu_{2i}{}^{3}|A_{i}=0\right)$$

### 3.3 Data sources and sampling

**Figure 2:**
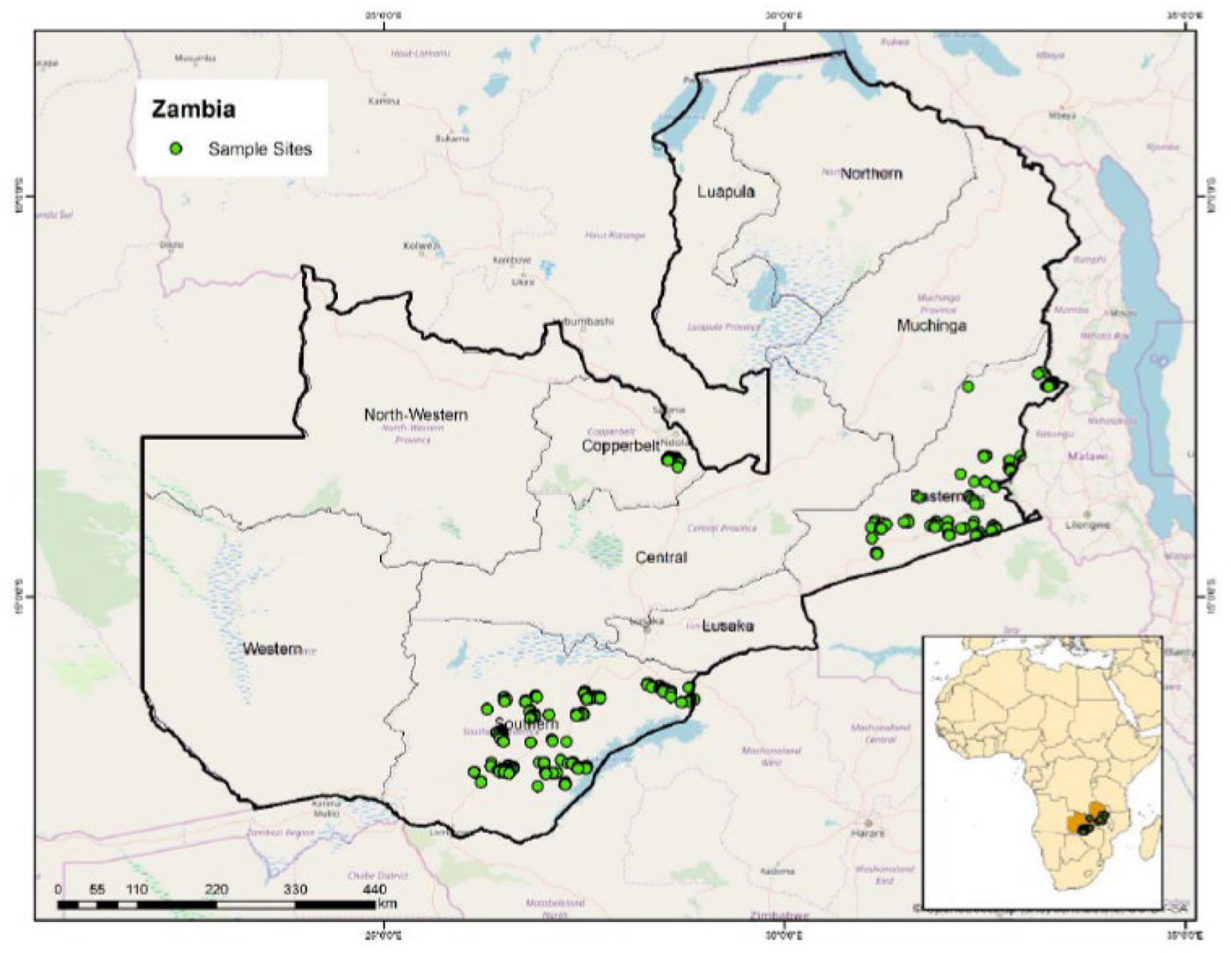
Location of surveyed households, Zambia

This study uses household survey data collected by a team led by the International Maize and Wheat Improvement Center (CIMMYT) from November to December 2015 in three maize producing provinces (Eastern, Southern, and Copperbelt) of rural Zambia (Figure 2). The survey covered a representative sample of 1100 households randomly selected in 11 districts using a three-stage sampling procedure. The sampling strategy ensured selection of equal number of households in each district (100 households in each) sampled as presented in Table II. The districts covered include; Masaiti in Copperbelt province, Chadiza, Chipata, Katete, Lundazi and Petauke in Eastern province, Choma, Kalomo, Monze, Siavonga and Sinazongwe in Southern province. The stages involved: identification of camps, selection of villages and subsequent sampling and selection of households for survey interviews. First, two agricultural camps in each district were purposively selected. Second, out of the 30-40 villages that are located in these camps, five villages were selected using a simple random sampling. Third, using the village headmen and Camp Agricultural Committee chairpersons, about ten households were systematically sampled from each village. Three households were later dropped during data analysis. Our sample design implies Copperbelt only makes up 8.9 per cent of the total sample, with the remainder (45.5 per cent each) split between Eastern and Southern.

To successfully implement this survey, the Zambia Agricultural Research Institute (ZARI), Ministry of Agriculture and Livestock (MAL) and village headmen and camp agricultural committee chairpersons provided support to identify areas, camps and households for the survey. A structured household questionnaire was used to collect data using face-to-face interviews technique, administered by well-trained enumerators after the pre-test exercise. The survey instrument was well designed – consisted of 14 modules that captured detailed information on a range of variables at both household and plot level. However, only few modules relevant to the objectives of this study were used in this study.

**Table I:** Conditional expectations, treatment and heterogeneity effects

Sub-groups	Decision stageTo adopt	Not to adopt	Treatment effects
Farm households that adopted DTMVs	*E*(*Y*_1*i*_|*A*_*i*_ = 1)	*E*(*Y*_2*i*_|*A*_*i*_ = 1)	*ATT*
Farm households that did not adopt DTMVs	*E*(*Y*_1*i*_|*A_i_* = 0)	*E*(*Y*_2*i*_|*A_i_* = 0)	*ATU*
Heterogeneity effects	*BH*_1_	*BH*_2_	*TH*

**Table II:** Number of households sampled in each province, Zambia

Name of province	No. of districts	No. of households
Copperbelt	1	98
Eastern	5	500
Southern	5	499
Total	11	1097

## 4 Results and discussions

### 4.1 Descriptive statistics

**Figure 3:**
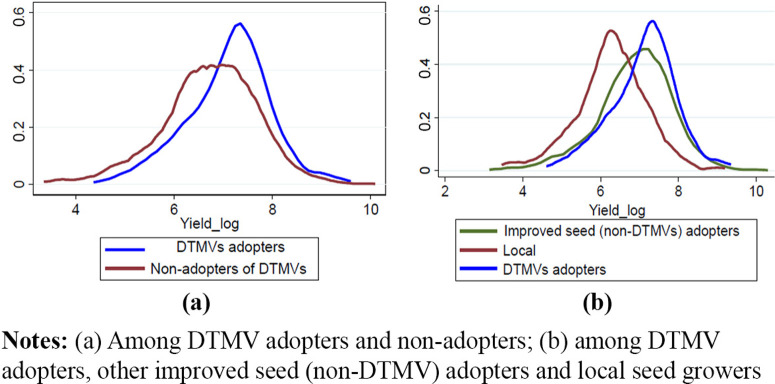
Distribution of maize yields

The major outcome indicator was maize yield. The distribution of maize yield between adopters of DTMVs and non-adopters using kernel estimates in Figure 3(a) and Figure 3(b) reveals that the average maize yield was slightly higher among DTMVs adopters in both cases, followed by improved non-DTMVs seed adopters and finally those planting local seed. Besides, a more left-skewed (negative) distribution on non-adopters of DTMVs and improved seed adopters as compared to DTMVs adopters is evident in Figure 3(b) signifying that the skewness of maize yield was lower among adopters. The Kolmogorov– Smirnov test for equality of distribution functions demonstrated that the two distributions between DTMVs adopters and non-adopters are different.

Tables III and IV present the descriptive statistics for all sampled households by DTMVs adoption status. Results indicate that 20 per cent of households had adopted at least one DTMV in 2015 with relative adoption being highest in Copperbelt province (37 per cent), about average in Eastern province (21 per cent) and relatively lowest in Southern province (16 per cent). The share of maize plots with DTMVs was slightly lower (13.5 per cent).

Only 19 per cent of households were headed by women. The mean age of household heads was 47 years. Households consisted of an average of 7 members with adopters having somewhat larger households. The average farm size was 4 ha, where maize (1.8 ha) occupied approximately 45 per cent of the average farm land and with adopters cultivating more maize area. Livestock keeping was also a key economic activity with households having an average of five tropical livestock units (TLU).

Nearly 77 per cent of households reported to have experienced drought in 2015. Concerning social capital, the majority of households (88 per cent) were members in at least one informal association, being more common for adopters, which may entail diverse benefits, notably access to information and influence. On information access, most households (70 per cent) obtained information on weather and rainfall. However, only few households (44 per cent) had access to information regarding new maize varieties. This underscores the need for a more concerted effort for DTMV awareness creation in the farming communities.

Male-headed households owned the majority of plots (73 per cent), and about 80 per cent of households reported using in-organic fertilizers at least in one of their plots. DTMV adopters used a significantly higher amount of fertilizers as compared to non-adopters. The mean average amount of basal dressing fertilizer (D-compound) used by a household was 162 kgs, while the topdressing fertilizer (urea) was 158 kgs.

**Table III:** Percentage of households growing at least one DTMV and per cent of plots under DTMVs, Zambia

Provinces	Percentage of households (within study region)	Percentage of plots (within study region)
Copperbelt	37	30
Eastern	21	17
Southern	16	8
Total	20	13.5

**Source:** Survey,2015

**Table IV:** Descriptive statistics by DTMV adoption status, Zambia

Variable	Full sample (*N* = 1097)	Adopters (*N* = 221)	Non-adopters (*N* = 876)	Mean difference
Household size	6.9	7.2	6.9	0.39^*^
Age of household head (years)	46.5	46.3	46.5	-0.2
Sex (1 = male, 0 = female)	0.81	0.84	0.81	0.039
Education of household head	6.06	6.66	5.9	0.76^**^
Distance to the nearest maize seed market (min)	70	80	68	12^**^
Wall material – wood and mud, reeds and bamboo, cement and stones,	0.72	0.77	0.70	0.07^**^
blocks or bricks (1 = yes)				
TLU of livestock^a^	4.5	5.1	4.4	0.67
Farm size (ha)	4.04	4.27	3.99	0.28
Maize area (ha)	1.8	2.2	1.7	0.5^***^
Dry spell in 2015 (1 = yes)	0.77	0.8	0.76	0.04
Obtain information on rainfall and weather (1 = yes)	0.7	0.73	0.7	0.03
Obtain information on new maize varieties (1 = yes)	0.44	0.43	0.44	-0.01
Member in informal association (1 = yes)	0.88	0.93	0.87	0.06^***^
Labor (Man-days)	85	101.5	81.3	20.2^*^
Hired labor use (1 = yes)	0.58	0.7	0.56	0.14^***^
Inorganic fertilizer use (1 = yes)	0.8	0.94	0.76	0.18^***^
D-Compound fertilizer (kgs)	162	254	139	115^***^
Urea (kgs)	158	250	135	114^***^
Pesticide use (1 = yes)	0.11	0.15	0.1	0.05^**^
Cover crop use (1 = yes)	0.16	0.15	0.16	-0.01
Intercropping (1 = yes)	0.67	0.62	0.68	-0.06
Good soil (1 = yes)	0.3	0.36	0.29	0.07^**^
Fair Soil (1 = yes)	0.4	0.45	0.39	0.06
Poor soil (1 = yes)	0.44	0.42	0.45	-0.03
Male-owned plots (1 = yes)	0.73	0.78	0.72	0.06^*^
Female-owned plots (1 = yes)	0.23	0.19	0.24	-0.05^*^
Joint-owned plots (1 = yes(	0.022	0.027	0.02	0.007
Maize yield Kg/ha	1374	1696	1292	403.8^***^
Maize grain sold (kgs)	929	1817	705	1111^***^
Maize self-sufficient (1 = yes)	0.7	0.78	0.68	0.10^***^

^***^Notes:*p* < 0.01

^**^p < 0.05

^*^p < 0.1.

^a^Total livestock units were calculated using the following conversion factors (cattle = 0.7, sheep and goats = 0.1, chicken = 0.01 and horses/donkeys = 0.8. www.fao.org/wairdocs/ilri/x5443e/x5443e04.htm

Source: Survey, 2015

The relatively high fertilizer application rates in Zambia (compared to SSA) are likely associated with the government fertilizer subsidy programs (FISP). Yet, while fertilizer use is expected to have a positive yield impact, the average yields registered by interviewed households are still low (1.3 tons per ha). This result suggests that although fertilizer use is important in soil fertility management and crop production, fertilizer use efficiency in Zambia is low. Consistent with this notion, Xu et al. (2009) and FAO (2015a, 2015b) suggest that farmers’ ability to acquire fertilizer on time has a strong positive effect on maize yield response to fertilizer and decreases the probability of low yields. However, it is noted that in Zambia subsidized fertilizers under government programs have often been distributed late (Xu *et al*., 2009). Furthermore, Burke et al. (2017) indicate that most of the soils in Zambia are naturally acidic (low PH), thus considerably limiting Zambian maize yields. Soil acidity and basal fertilizer application have significant interactions suggesting that productivity of phosphoric fertilizer is seriously limited by soil acidity and top dressing fertilizer is more effective than basal on Zambian soils (Burke *et al*., 2017; Burke, 2012).

Generally, labor use and hired labor was high among adopters (102 person-days and 70 per cent, respectively). Intercropping was a common practice by 67 per cent of households while pesticide use and cover crops were uncommon (by 11 and 16 per cent of households, respectively). Even though the DTMV adopters in this study had a higher level of input use, past studies suggest DTMVs varieties to have similar agronomic, labor requirements and seed costs as other (non-DTMVs) commercial varieties, whereas some are also nitrogen-use efficient (Fisher *et al*., 2015). Kostandini et al. (2013) further express that DTMVs have superior maize yields and yield stability performance than other improved non-DTMVs even with little or no fertilizer use[3].

### 4.2 Ordinary least squares (OLS) results

In first instance, a generalized linear model (OLS) was used to determine the effect of DTMV adoption on mean yield, variance and skewness. OLS is the simplest approach to investigate the effect of adoption on food production that includes a dummy variable equal to 1 if the farm household adopted and 0 otherwise (Di Falco *et al*., 2010). Control variables used in the OLS model for our study include household and farm characteristics that were assumed to affect farmer’s adoption decision and also productivity, risk, food security and poverty (Table V).

OLS results (Table V) suggest that DTMV adoption had a positive statistically significant effect on maize productivity and skewness, with DTMV adoption increasing maize yields by 23 per cent and reducing yield variance by 21 per cent and exposure to downside risk by 18 per cent. These results illustrate that DTMV adoption can serve as productivity-enhancing as well as serve as insurance for farmers by reducing yield variability and minimizing the risk of crop failure.

Although the OLS results show that there was an increase in maize yield and reduction in risk for DTMV adoption, these results are not reliable because the OLS model yields biased and inconsistent estimates. In this case, the approach assumes that DTMV adoption is exogenously determined while it is a potentially endogenous variable. Moreover, as expressed by Di Falco et al. (2010), the OLS estimates do not explicitly account for potential structural differences between the production function of farmers who adapted to climate change and the production function of farmers that did not adapt.

### 4.3 Endogenous switching regression results

To address the limitations identified with OLS model above and to test the robustness of the results, we also used the ESR model that accounts for both observable and unobserved sources of heterogeneity between adopters and non-adopters.

**Table V:** titleOLS estimates of the effects of DTMV adoption on mean, variance and skewness of maize yields, Zambia

Variable	Maize yield	Variance	Skewness
DTMVs adoption	0.227^***^(0.071)	0.209 (0.147)	0.182^**^(0.083)
Household size	0.015^*^(0.008)	0.002 (0.017)	0.018^*^(0.009)
Gender	0.056 (0.101)	0.043 (0.209)	0.039 (0.119)
Age in years	0.002 (0.002)	0.004 (0.004)	0.001 (0.002)
Received information on new maize varieties	0.055 (0.056)	0.269^**^(0.117)	0.009 (0.066)
Other farmers as source of new information			
on new maize	0.064 (0.067)	0.204 (0.139)	0.021 (0.079)
Hired labor use	0.138^***^(0.048)	0.109 (0.100)	0.142^**^(0.057)
Pesticide use	0.199^***^(0.066)	0.045 (0.138)	0.206^**^(0.078)
Membership in groups	0.173^**^(0.088)	0.079 (0.182)	0.184^*^(0.103) Obtained information on rainfall
Obtained information on rainfall	0.027 (0.052)	–0.219^**^(0.110)	0.000 (0.062)
Good soil fertility	0.150^**^(0.063)	0.106 (0.131)	0.140^*^(0.074)
Fair soil fertility	0.152^***^(0.058)	0.049 (0.120)	0.158^**^(0.068)
Plot managed by male	0.051 (0.094)	–0.019(0.195)	0.026 (0.110)
Occurrence of dry spell in 2015	–0.232^***^(0.058)	–0.035 (0.121)	–0.209^***^(0.068)
Intercropping	–0.059 (0.046)	0.098 (0.096)	–0.034 (0.054)
No erosion on plots	0.067 (0.083)	0.075 (0.172)	0.025 (0.097)
Flat plot slope	0.046 (0.063)	0.015 (0.130)	0.017 (0.074)
Moderate plot erosion	0.091 (0.070)	0.197 (0.144)	0.049 (0.081)
Labor (log)	0.039 (0.024)*	0.099^**^(0.049)	0.032 (0.028)
Inorganic fertilizer (log)	0.109^***^(0.010)	0.008 (0.021)	0.102^***^(0.012)
Eastern province	–0.024 (0.098)	0.255 (0.204)	–0.036 (0.116)
Southern province	0.186^*^(0.096)	0.328^*^(0.199)	0.172 (0.112)
Observations (plots)	1876	1876	1876

**Notes:** Parenthesis figures indicate the standard errors;

^***^p < 0.01

^**^p < 0.05

^*^p < 0.1

#### 4.3.1 Determinants of adoption and maize yields

Results of the determinants of DTMV adoption and maize yield for both adopters and non-adopters are presented in Table VI. Seed and fertilizer subsidies were used as instrumental variables as shown in the selection equation. The results confirm that indeed the instrument (seed subsidy) was relevant, as it had a positive and statistically significant effect on the probability of adopting DTMVs. Other significant factors favoring adoption in the selection equation include fertilizer and labor use, hired labor use and flatter plots with moderate erosion. In contrast, reliance on informal sources of information on new maize varieties reduced the probability of adoption.

Maize yield for both adopters and non-adopters was strongly influenced by fertilizer use in line with expectations. But whereas household size had a favorable effect on maize yields for adopters, the reverse was true for non-adopters, for whom hired labor use favorably affected yields. Age had a negative effect for adopters, whereas other factors influenced yields for non-adopters (Table VI). Perhaps most interestingly, the occurrence of a dry spell in 2015 reduced yields only for non-adopters, whereas having no yield effect for adopters.

#### 4.3.2 Effect of adoption of drought-tolerant maize varieties on mean yield, variance and skewness

We used ESR to compare the distribution of maize mean yield, variance and skewness with and without DTMV adoption (Table VII).We adopted one of the most efficient estimation method (the movestay command) recommended by Lokshin and Sajaia (2011) due to the fact that it enables the implementation of the full information ML method to simultaneously estimate binary and continuous parts of the model to yield consistent standard errors. This counterfactual analysis method enabled comparisons of the expected outcome indicators under the actual and counterfactual cases that the farm household adopted DTMVs Estimations of treatment and heterogeneity effects enabled the understanding of the differences in maize productivity and risk reduction between farm households that adopted DTMVs (ATT) and those that did not adopt (ATU) DTMVs.

**Table VI:** titleDTMV adoption and maize yields determinants, Zambia

Variable	DTMV adoption (selection equation)		Maize yield determinants			
			Adopters		Non-adopters	
	Coefficient	Z value	Coefficient	Z value	Coefficient	Z value
Education	0.008 (0.012)	0.63	0.018 (0.018)	0.98	0.009 (0.008)	1.10
Age in years	0.003 (0.003)	0.80	0.009 (0.005)	1.83^*^	0.001 (0.002)	0.54
Household size	0.002 (0.004)	0.11	0.071 (0.023)	3.15^***^	0.023 (0.009)	2.63^***^
Membership in groups	0.081 (0.157)	0.52	0.027 (0.235)	0.12	0.202 (0.094)	2.15^**^
Other farmers as a source of information on new maize	0.234 (0.102)	2.28^**^	0.219 (0.179)	1.23	0.044 (0.063)	0.71
Occurrence of dry spell in 2015	0.104 (0.101)	1.03	0.213 (0.15)	1.41	0.247 (0.063)	3.92^***^
Inorganic fertilizer (log)	0.083 (0.02)	4.22^***^	0.17 (0.049)	3.45^***^	0.099 (0.012)	8.32^***^
Good soil fertility	0.054 (0.092)	0.58	0.079 (0.133)	0.60	0.053 (0.061)	0.86
Hired labor use	0.166 (0.084)	1.98^**^	0.084 (0.147)	0.57	0.139 (0.053)	2.62^***^
Pesticide use	0.039 (0.112)	0.35	0.241 (0.163)	1.47	0.146 (0.073)	1.99^**^
No erosion on plots	0.155 (0.106)	1.46	0.130 (0.168)	0.78	0.013 (0.071)	0.18
Labor (log)	0.156 (0.040)	3.87^***^	0.055 (0.083)	0.66	0.04 (0.027)	1.48
Plot managed by male	0.024 (0.104)	0.23	0.097(0.154)	0.63	0.106 (0.065)	1.62^*^
Flat plot slope	0.353 (0.127)	2.78^***^	0.041 (0.229)	0.18	0.037 (0.084)	0.44
Moderate plot erosion	0.197 (0.114)	1.73^*^	0.085 (0.188)	0.45	0.086 (0.069)	1.25
Seed subsidy	0.206 (0.096)	2.16^**^				
Fertilizer subsidy	0.061 (0.092)	0.66				
Wald chi2	37.1^***^					
N (no. of plots)	1876					

**Notes:** Figures in parenthesis indicate the standard errors;

^***^p < 0.01

^**^p < 0.05

^*^p < 0.1

**Table VII:** ESR Estimates of the effect of DTMV adoption on mean, variance and skewness of maize

Outcome variables	Household type and treatment effect	Decision stage			
		To Adopt	Not to adopt	Effect on adoption	Change (%)
Average maize yield	Adopters (ATT)	7.10	6.17	0.93^***^	15
	Non-adopters (ATU)	6.64	6.75	-0.11^***^	-1.6
	Heterogeneity effect	0.46	0.58	1.03	
Average variance	ATT	12.80	20.75	–7.95^***^	–38
	ATU	12.77	13.01	–0.24^***^	–1.8
		0.03	7.74	–7.71	
Average skewness	ATT	14.69	10.84	3.85^***^	35.5
(downside risk)	ATU	13.15	14.32	-1.16^***^	-8.1
	Heterogeneity effect	1.54	–3.47	5.01	

^***^Note:*p* < 0.01

Table VII reveals that DTMV adoption increased average yield significantly by 15 per cent. This implies that maize yield among DTMVs adopters would have declined by 15 per cent if they had not adopted. Besides, the positive transitional heterogeneity effect reveals that the effect is larger for the farm household that actually did adopt compared to those that did not adopt DTMVs. These strong positive impacts of DTMVs on maize yields among the adopters implies that the ongoing continuous concerted efforts on awareness and dissemination of DTMVs by private and public institutions can greatly contribute to increased food security and resilience of households to drought in Zambia.

With regard to variance and skewness of maize yield, ATT and ATU results indicate that the DTMV adoption significantly reduced yield variability and exposure to downside risk. Particularly, DTMV adoption reduced the variability of maize yield by 38 per cent, suggesting that the yield variance encountered by DTMVs adopters would have increased by 38 per cent if they had not adopted DTMVS. Similarly, DTMV adoption reduced the risk of crop failure by 36 per cent. The transitional heterogeneity effects on variance and skewness were negative and positive, respectively, indicating that adopters in both cases were better off than non-adopters.

ESR results on maize productivity and production risk factors (both yield variance and downside risk), suggest that, DTMVs have a win-win outcome because they do not only increase yields but also are risk reducing. Our results on DTMV effects on maize productivity and risk in Zambia were consistent with those reported in Nigeria by Wossen et al. (2017). This is an important characteristic of DTMV as risk and risk aversion influences management decisions of farmers. Other agricultural technologies often have mixed results on productivity and production risks. Abro (2018) did find that increased tillage intensity had complementary benefits on both productivity and risk exposure for wheat farmers in Ethiopia. On the other hand, Zhu (2018) observed that fertilizer use increased both yield and variances; intercropping decreased yield variance but did not increase expected returns, whereas combined adoption of the two led to higher expected returns albeit with variations by subgroups of farmers. Due to the risk-averse nature of smallholder farmers, one may find high rates of adoption of relatively unprofitable technologies with low risk, whereas profitable technologies with high risk may only show limited adoption. Hardaker et al. (2015) indeed affirm that risk-averse people may be willing to forgo some expected output for a reduction in risk, the rate of acceptable trade-off depending on how risk-averse that person is. Mukasa (2018) considering four moments of production (mean, variance, skewness and kurtosis) and the probability of adopting several modern inputs (including, improved seeds, chemical fertilizer and pesticides) revealed that most modern inputs are risk reducing though sometimes at great cost to farmers.

Taking into account different adaptation strategies, Di Falco and Veronesi (2018) noted that even though past climate change adaptation decreased current downside risk exposure, households that did not adapt in the past would benefit the most from adaptation in terms of reduction in downside risk exposure. Similarly, Kassie et al. (2015) revealed that adoption of sustainable intensification practices in Malawi had complementary benefits in increasing food security (in terms of maize yields) and reducing both probability of crop failure and risk cost. They particularly pointed out that adoption of crop diversification in combination with minimum tillage resulted in greater increases in food security and reduction in downside risk.

From the above discussion, it can be deduced that adaptation is one of the successful risk management strategies that make farmers resilient to changing climatic conditions. DTMVs are particularly promising given there win-win outcome and potential use as a risk management strategy. Still, given the multiple overlapping production challenges that farmers face, adoption of multiple agricultural innovations (including DTMVs, alongside better agronomic practices) may well provide further complementary benefits in production, food security and risk mitigation (Manda *et al*., 2016; Kassie *et al*., 2015; Gebremariam, 2018). Therefore, institutions involved in agricultural technology dissemination should consider including DTMVs in their input packages and be on the forefront to help farmers in building resilience to drought as well as improve food security and poverty reduction.

## 5 Conclusions and implications

A central question in most adoption studies is why different farmers in a specific locality adopt a specific type of technology(s) at different moments and magnitudes. Most SSA smallholders aim to improve their food security status and sustain their livelihoods. Therefore, any given new agricultural technology should be effective in terms of either productivity, profitability, risk reduction, welfare, food security or poverty reduction. In view of this, the effects of adopting DTMVs on productivity and production risks were analyzed in this study using household and farm level data from rural Zambia. The results from the ESR model revealed significant yield gains and production risk reduction among DTMVs adopters. Specifically, DTMV adoption increased maize yields by 15 per cent. Furthermore, DTMVs reduced the level of yield variability by 38 per cent and reduced exposure to downside risk by 36 per cent.

These findings have some policy implications. First, given the myriad of challenges faced by smallholder farmers in SSA including climate change, the role of risk in technological options is integral for effective agricultural policies and should never be overlooked. Research and implementing institutions should take into consideration multiple and higher-order moments of production distribution beyond the mean when designing intensification and innovation policies so as to meet farmers’ different preferences. In particular, design and promotion of effective drought adaptation strategies that address both yield gains (and profitability) and risk reduction in terms of yield variability and downside risk are critical for improved food security and livelihoods in the face of an already highly variable and changing climate. Most of the farmers are risk averse and at the same time conscious about the expected returns. Therefore, when such win-win technologies are actually available and promoted effectively, farmers will most likely be motivated to embrace them potentially stimulating adoption and impacts.

In the end, the adoption rates of improved technologies and their enhanced productivity and risk reduction impacts largely depend on how the farmers access relevant information about the technology. Imperfect information about the new technology as a result of lack of information, misinformation or ineffective knowledge sharing pathways can be a severe limitation slowing the adoption and diffusion process. Therefore, intensified awareness efforts on seed availability, favorable climatic and agronomic requirements, performance characteristics and other special attributes are essential for any seed-based technology. Information can be effectively disseminated through a combination of different pathways. Pathways can be “effective change agents” and can greatly influence the probability of adoption especially if they entail “seeing is believing” within the local context and are participatory in nature. Such methods include on-farm demonstration plots, farmer field days, exhibitions and agricultural shows distribution of sample seed packs, regular extension-farmer visits, print and electronic media among others.

Finally, awareness raising efforts should ensure seed availability so that when farmers are made aware and want the seed, it can be adequately, easily and timely accessed. Public– private partnership efforts need to provide synergistic and complementary support to effectively deploy both market and non-market-based approaches to further scale out drought-tolerant maize varieties in Zambia by addressing issues related to seed demand, accessibility and availability.

## Notes

1 http://maize.org/why-maize/

2 Available at:http://dtma.cimmyt.org/index.php/about/background

3 More information on DTMVS are available at:http://dtma.cimmyt.org/index.php/about/background
